# Mice lacking Programmed cell death-1 show a role for CD8^+^ T cells in long-term immunity against blood-stage malaria

**DOI:** 10.1038/srep26210

**Published:** 2016-05-24

**Authors:** Joshua M. Horne-Debets, Deshapriya S. Karunarathne, Rebecca J. Faleiro, Chek Meng Poh, Laurent Renia, Michelle N. Wykes

**Affiliations:** 1 The QIMR Berghofer Medical Research Institute, Infectious Disease Programme, Queensland, 4029, Australia; 2 The School of Medicine, University of Queensland, Brisbane 4072, Australia; 3 Singapore Immunology Network, Agency for Science, Technology and Research (A^*^STAR), 136648, Singapore; 4 Department of Microbiology, Yong Loo Lin School of Medicine, National University of Singapore, 117597, Singapore

## Abstract

Even after years of experiencing malaria, caused by infection with *Plasmodium* species, individuals still have incomplete immunity and develop low-density parasitemia on re-infection. Previous studies using the *P. chabaudi* (*Pch*) mouse model to understand the reason for chronic malaria, found that mice with a deletion of programmed cell death-1 (PD-1KO) generate sterile immunity unlike wild type (WT) mice. Here we investigated if the mechanism underlying this defect during acute immunity also impacts on long-term immunity. We infected WT and PD-1KO mice with *Pch*-malaria and measured protection as well as immune responses against re-infections, 15 or 20 weeks after the original infection had cleared. WT mice showed approximately 1% parasitemia compared to sterile immunity in PD-1KO mice on re-infection. An examination of the mechanisms of immunity behind this long-term protection in PD-1KO mice showed a key role for parasite-specific CD8^+^ T cells even when CD4^+^ T cells and B cells responded to re-infection. These studies indicate that long-term CD8^+^ T cell-meditated protection requires consideration for future malaria vaccine design, as part of a multi-cell type response.

Malaria, caused by parasites of the genus *Plasmodium,* is responsible for approximately 225 million infections and 584,000 deaths annually, mostly of children under 5 years of age^1^. Humans can be infected with five known species of *Plasmodium* but *P. falciparum* (*Pf*) infections are the most prevalent and leading cause of malaria-related mortality, although *P. vivax* (*Pv*) and *P. knowlesi* can also produce severe disease^2^. Development of an effective vaccine against *Pf* malaria has been a target of much research, yet the best vaccine candidate to date, RTS,S/AS01E had an overall efficacy of 16.8% over a 4-year period^3^.

In endemic settings, naturally-acquired immunity to *Pf* confers only partial non-sterile protection that protects individuals from severe disease^4^. This protection only results after repeated infections but wanes in absence of continued exposure^4^. The components of the immune system responsible for killing *Plasmodium* parasites remain unclear, although antibodies are known to have a key role in controlling blood-stage infections^5^. Protection in adults has traditionally been attributed to the acquisition of a repertoire of specific, protective antibodies directed against polymorphic and conserved target antigens^6^. In addition, studies in rodent models^7^ and in infected patients^8^ have found that parasite-specific CD4^+^ T cells were not well maintained following malaria. Complete protection against homologous *Pch* re-infections can occur up to 8 weeks following primary infection in mice^9^. However, between 10 and 18 weeks, protection wanes to a reduction in peak parasitemia and duration of infection rather than rapid elimination of parasites^10^.

PD-1 has been implicated in “exhaustion” of T cells which is characterized by poor effector function and sustained expression of inhibitory receptors, resulting in a transcriptional state distinct from that of functional effector or memory T cells, which together prevents optimal control of infections and tumors. Recent studies implicated PD-1 in modulating immunity against malaria by showing expression of this molecule on mouse^11^,^12^,^13^ and human^14^,^15^ T cells during acute infections. Most significantly, *in vivo* blockade of PD-1 ligand 1 (PD-L1) and the inhibitory receptor LAG-3 in mice accelerated the clearance of non-lethal *P. yoelii* malaria^14^ while blockade of PD-L1 *per se* augmented experimental *P. berghei* cerebral malaria^12^. In earlier studies, we showed a PD-1-mediated loss of numbers and functional capacity of parasite-specific CD8^+^ T cells during the acute phase of *Pch* malaria, which exacerbated acute infections and caused chronic disease^13^. Here we explored if the PD-1-mediated loss of immunity during acute malaria could impact on long-term immunity against malaria. Accordingly, we infected C57BL/6 (WT) and PD-1KO (on C57BL/6 background) mice with non-lethal *Pch* which causes chronic malaria in WT mice. After the clearance of primary infection, mice were rested for 15 or 20 weeks to allow all primary immune cells to subside (~10 weeks)^16^ and then re-infected to measure the role of memory CD4^+^ and CD8^+^ T cells and B cells in long-term protection as assessed by the ability to control blood-stage parasitemia. To understand the mechanism of protection, responses by memory cells were measured within 5 days after re-infection, prior to the development of new primary response which take 7–10 days. These studies show a previously unknown crucial role for CD8^+^ T cells and IFN-γ in long-term protection against malaria.

## Results

### PD-1 reduces long-term protection against murine malaria

To assess long-term protection against malaria in mice, WT and PD-1KO mice were infected with *Pch* parasitized red cells (pRBC) and after 40 days when patent parasitemia had cleared, mice were rested for 140 days (20 weeks) to allow primary immune responses to subside. Mice were then re-infected with *Pch* and parasitemia was monitored to assess protection by long-lived memory cells.

**Figure 1:**
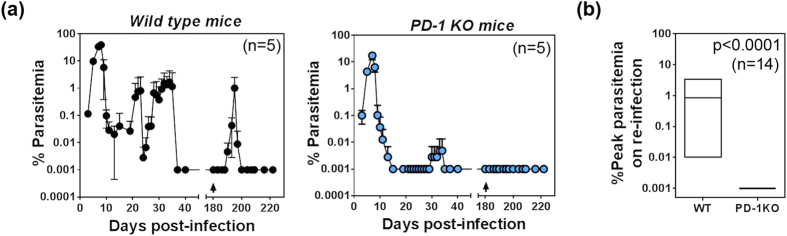
*P. chabaudi AS* malaria induces PD-1-dependent loss of protection. **(a)** Mean percent parasitemia during a typical course of infection in cohorts of WT and PD-1KO mice infected with 10^5^ *Pch* pRBC, rested for 20 weeks after the clearance of patent parasitemia (~40 days) and re-infected (arrow) with the 10^5^ *Pch* pRBC (n = 5 of 14 total mice in 3 experiments). Error bars represent SD. **(b)** Floating box graph of maximum and minimum peak parasitemia in WT and PD-1KO mice following re-infection with line at mean (n = 14).

Previously infected WT mice developed a mean peak parasitemia of ~1% after 15 days following re-infection, unlike primary infections which peak after 8 days with ~38.8% peak parasitemia (Fig. 1a; left panel). This indicated that immunological memory did offer substantial protection from homologous re-infection by delaying and reducing peak parasitemia and duration of the second infection. In contrast, PD-1KO mice had reduced primary peak and recrudescent parasitemia compared to WT mice (note log scale on Fig. 1), < 30% of the PD-1 KO mice developed chronic infections, and recrudescent parasitemia levels in these mice were > 100-fold lower than those in the WT mice as seen previously^13^. Following homologous re-infection 140 days after the clearance of the primary/recrudescent infections, in 3 independent experiments (n = 14 total), PD-1KO mice remained completely free parasitemia within the detection limit of 0.001% by microscopy (Fig. 1a; right panel). Transfer of 200 μl blood from 5 of these mice to naïve C57BL/6 mice did not transfer the infection indicating sterile immunity. The difference between peak parasitemia following challenge of WT and PD-1KO mice was highly significant (p < 0.0001; Fig. 1b). Overall, these data showed that WT mice infected with *Pch* malaria do have substantial protection against re-infection. However, by comparison to PD-1KO mice, immunity is incomplete in WT mice because PD-1 contributes to the loss of sterile immunity.

### PD-1 does not significantly alter long-term B cell responses

To establish whether PD-1 signaling modified long-term B cell responses, cohorts of WT or PD-1KO mice were infected with *Pch* pRBC, rested for 15 weeks after the clearance of patent primary parasitemia (~35–40 days) and then re-infected with the same dose of *Pch.* Sera from these mice were collected a day before and 5 days after re-infection, and tested by ELISA for parasite-specific IgG antibody titers. We examined responses after 5 days to give the memory response sufficient time to respond to the new infection but before new primary responses are initiated.

**Figure 2:**
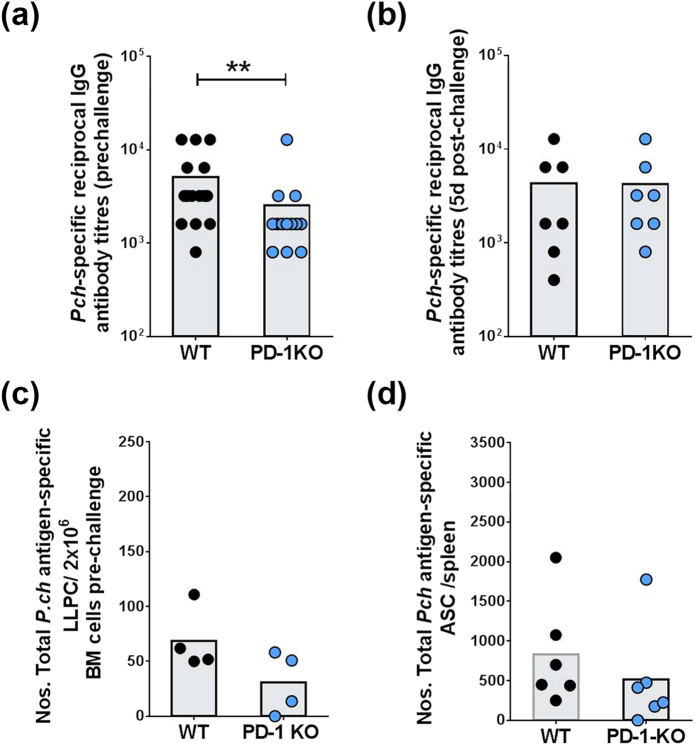
*P. chabaudi AS* malaria induces PD-1-dependent loss of protection independent of B cell responses. *Pch*-specific reciprocal IgG antibody titers were measured **(a)** 1 day before (pre-challenge) and **(b)** 5 days after re-infection (post-challenge), by ELISA. **(c)** Numbers of parasite-specific plasma cells per 2 × 10^6^ bone marrow (BM) cells prior to re-infection by ELISPOT assay. **(d)** Numbers of total *Pch*-antigen-specific ASC per spleen 5 days after re-infection by ELISPOT assay with pre-infection levels subtracted. Bars show mean values with individual data points from pooled experiments. *P* values were determined using the non-parametric Mann-Whitney *U* test on pooled data from replicate experiments. (**p < 0.005 between WT and PD-1 KO groups).

WT mice had significantly higher titers of antibody than PD-1KO mice before re-infection (Fig. 2a). This most likely reflects the observation that WT mice have recrudescent infections for 2.5 to 3 months after primary infection^10^ capable of re-stimulating antibody responses unlike PD-1KO mice which develop sterile immunity after the primary infection^13^. However, after re-infection, both groups had similar titers (Fig. 2b). Examination of bone marrow (BM) from infected and rested mice before re-infection by an ELISPOT assay was undertaken to quantify long-lived plasma cells (LLPC) which would secrete parasite-specific IgG antibody independent of re-infection. There were no significant differences between WT and PD-1KO mice although the latter group showed a trend for fewer antibody-secreting LLPC (Fig. 1c).

To quantify memory B cells (MBC), which differentiate into antibody secreting cells (ASC) to secrete parasite-specific IgG antibody, we next examined spleen cells from mice without and 5 days after re-infection, by a parasite-specific ELISPOT assay for secreted antibodies. The number of ASC per spleen from MBC responses was calculated as the difference between ASC in mice with and without (mean of 3 mice) re-infections. There were no significant differences in numbers of total parasite antigen-specific ASC between WT and PD-1KO mice (Fig. 2d). These data indicated that anti-malarial MBC responses were not significantly affected by PD-1 signaling. The numbers of MBCs seen in WT mice were similar to those previously seen by vaccination with a malaria vaccine and did not increase in PD-1KO mice, indicating PD-1 cannot overcome BAFF -deficiency-mediated loss of ASCs^17^. Overall, these studies showed that improved protection seen in PD-1KO mice compared to WT mice (Fig. 1a) was not due to B cell responses as the latter had higher antibody titers prior to re-infection and similar numbers of LLPC and MBC.

### PD-1 does not alter CD4^+^ T cell responses in long-term anti-malarial immunity

**Figure 3:**
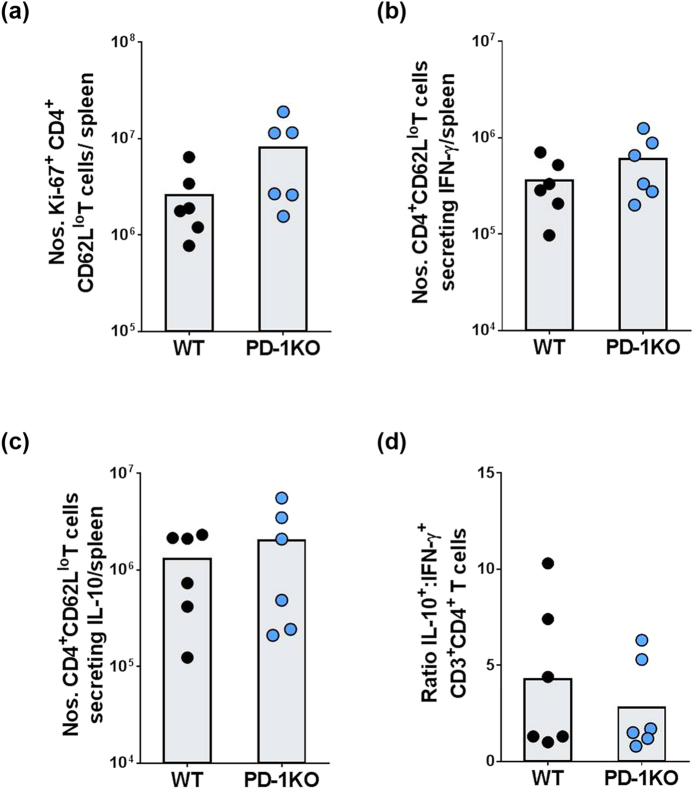
Phenotypic and functional analysis of CD4^+^ from *Pch*-infected WT and PD-1KO mice during long-term recall responses. WT and PD-1KO mice were infected with 10^5^ *Pch* pRBC, rested for 15 weeks after the clearance of patent parasitemia (~40 days), re-infected with 10^5^ *Pch* pRBC and spleen cells analyzed after 5 days post-infection. **(a)** Mean numbers of CD4^+^ CD62L^lo^ T cells expressing Ki-67 per spleen. **(b)** Mean numbers of CD4^+^ CD62L^lo^ T cells per spleen that secreted IFN-γ *ex-vivo*, as measured by a flow cytometry-based IFN-γ-secretion assay. **(c)** Mean numbers of CD4^+^ CD62L^lo^ T cells per spleen that secreted IL-10 *ex-vivo*, as measured by a flow cytometry-based IL-10-secretion assay. **(d)** Ratio of IL-10: IFN-γ- secreting CD4^+^ CD62L^lo^ T cells. Bars show mean values with individual data points from pooled experiments. Statistical significance determined using the non-parametric Mann-Whitney *U* test on pooled data from replicate experiments.

Next, we examined the role of PD-1 in CD4^+^ CD62L^lo^ effector memory T cell responses, 5 days following homologous re-infection as described above. We focused on Th1 responses, which are known to contribute to anti-malarial immunity^18^,^19^,^20^,^21^. There were no significant differences in numbers of recently proliferating (Ki67^+^; Fig. 3a), IFN-γ-secreting (Fig. 3b), or IL-10-secreting (Fig. 3c) CD4^+^ CD62L^lo^ T cells between WT and PD-1KO mice using flow cytometry based assays. The ratio of IL-10-secreting to IFN-γ secreting CD4^+^ T cells also was unchanged between WT and PD-1KO mice (Fig. 3d) indicating IL-10-mediated immuno-suppression was not responsible for less protection in WT mice. Overall, these studies showed that the difference in protection following re-infection between WT and PD-1KO mice (Fig. 1a) was not due to differences in CD4^+^ T cell responses.

### PD-1 inhibits parasite-specific CD8^+^ T cell responses to re-infection

**Figure 4:**
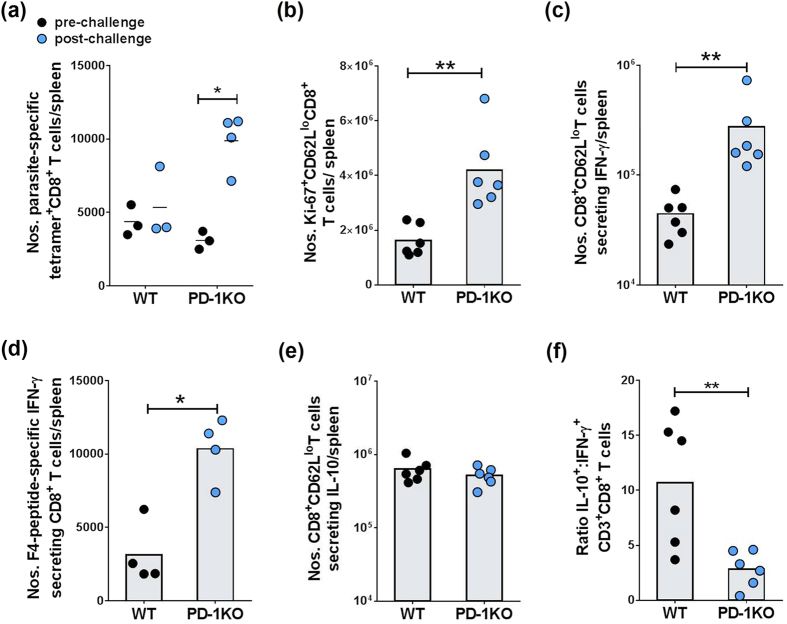
Phenotypic and functional analysis of CD8^+^ T cells from *Pch*-infected WT and PD-1KO mice during long-term recall responses. WT and PD-1KO mice were infected with 10^5^ *Pch* pRBC, rested for 15 weeks after the clearance of patent parasitemia (~40 days), re-infected with 10^5^ *Pch* pRBC and spleen cells analyzed after 5 days post-infection. **(a)** Mean numbers of parasite-specific F4-tetramer^+^ CD8^+^CD62L^**lo**^ T cells per spleen, without and 5 days after re-challenge. **(b)** Mean numbers of CD8^+^CD62L^lo^ T cells expressing Ki-67 per spleen. **(c)** Mean numbers of CD8^+^CD62L^lo^ T cells per spleen that secreted IFN-γ *ex vivo*, as measured by a flow cytometry- IFN-γ-secretion assay. **(d)** Mean numbers of parasite-specific CD8^+^ T cells per spleen that secreted IFN-γ in response to parasite-specific peptide F4 compared to cultures without peptide, as measured by ELISPOT assay. **(e)** Mean numbers of *ex-vivo* CD8^+^CD62L^lo^ T cells per spleen that secreted IL-10, as measured by a flow cytometry-based IL-10-secretion assay. **(f)** Ratio of IL-10: IFN-γ secreting cells CD8^+^CD62L^lo^ T cells. Bars show mean values with individual data points from pooled experiments. *P* values were determined using the non-parametric Mann-Whitney *U* test on pooled data from replicate experiments. (*p < 0.05; **p < 0.005 between WT and PD-1 KO groups).

To establish whether PD-1 signaling affected memory CD8^+^ T cells, we next labeled CD8^+^CD62L^lo^ T cells from mice with and without re-infection, with parasite-specific tetramers to quantify parasite-specific CD8^+^ T cells. While the re-infection had no significant effect on numbers of parasite-specific CD8^+^ T cells in WT mice, these numbers were increased by 3-fold in PD-1KO mice (Fig. 4a). This indicated that memory CD8^+^ T cells expanded in PD-1KO mice in response to re-challenge with the parasite but could not expand in WT mice, most likely due to PD-1-mediated exhaustion during acute malaria. We then more closely examined CD8^+^ T cell responses in re-infected mice. Firstly, CD8^+^ T cells were examined for recent proliferation as measured by Ki-67 expression by flow cytometry. Higher numbers of proliferating (Ki67^+^) CD8^+^CD62L^lo^ T cells were observed in PD-1KO mice compared to WT mice (Fig. 4b). Similarly, higher numbers of (CD62L^lo^) CD8^+^ T cells secreting IFN-γ both *ex vivo* (Fig. 4c) and in response to parasite-specific peptide *in vitro* (Fig. 4d) were detected in PD-1KO mice compared to WT mice. Numbers of IL-10-secreting CD8^+^ effector/memory T cells were similar in PD-1KO compared to WT mice (Fig. 4e). The overall ratio of IL-10 to IFN-γ secretion by CD8^+^ T cells was significantly reduced in PD-1KO mice compared to WT mice (Fig. 4f), consistent with better pro-inflammatory CD8^+^ T cell responses to re-infection. Overall, these data showed that effector memory CD8^+^ T cells secreting IFN-γ were associated with better protection against *Pch* malaria as seen in PD-1KO mice.

### PD-1 impedes long-term immunity by reducing CD8^+^ T cell- and IFN-γ-mediated protection

**Figure 5:**
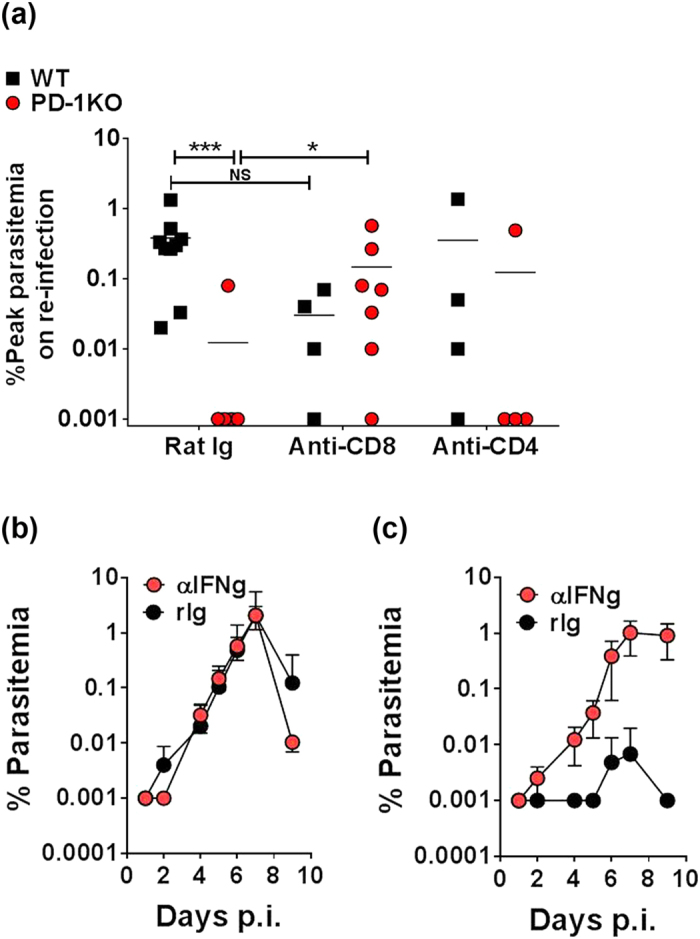
CD4^+^, CD8^+^ T cell and IFN-γ depletion show their contribution to protection during long term memory responses. WT and PD-1KO mice were infected with 10^5^ *Pch* pRBC, rested for 15 weeks after the clearance of patent parasitemia (~40 days), re-infected with 10^5^ *Pch* pRBC. Antibodies capable of depleting CD4^+^ or CD8^+^ T cells and IFN-γ or control Rat IgG (rIg) were administered on day -1 of re-infection and then every 2-3 days. **(a)** Scatter plots of peak parasitemia following re-infection in individual mice monitored for 10 days post-infection (p.i.), in WT (black square) and PD-1KO (red circles symbol) mice, following treatment with rIg, or the depletion of CD4^+^ T cells, or CD8^+^ T cells. Bars show mean of individual data points from 2 independent experiments. **(b,c)** Mean percent parasitemia in *Pch*-infected **(b)** WT mice and **(c)** PD-1KO mice, following depletion of IFN-γ compared to rIg treatment. The data represent one of 2 independent experiments (n = 6) with the mean percentage parasitemia ± SD. (NS: not significant; *p < 0.05; ***p < 0.001; between WT and PD-1 KO groups).

To address whether improved parasite control in PD-1KO mice was mediated by T cells, several cohorts of infected and rested WT and PD-1KO mice were depleted of CD4^+^ or CD8^+^ T cells with specific antibodies or treated with Rat Ig (rIg), 1 day before *Pch re-*infection and then every 3–4 days until days 12–14 p.i. Previous studies established the efficacy of this approach^13^. All WT mice treated with rIg (n = 9) had detectable parasitemia following re-infection and depletion of CD4^+^ or CD8^+^ T cells (n = 4) did not increase parasite burden (Fig. 5a). In comparison, only 1 of 7 PD-1KO mice treated with rIg had detectable parasitemia (Fig. 5a). Similarly, only 1 of 4 PD-1KO mice showed parasitemia on depletion of CD4^+^ T cells indicating limited contribution of these cells to long-term protection in the absence of PD-1. In contrast, 86% of PD-1KO mice depleted of CD8^+^ T cells had patent parasitemia on re-infection. Overall, CD8^+^ T cells were shown to control parasitemia at sub-patent levels in PD-1KO mice, following homologous re-infection of previously infected mice.

To determine if IFN-γ contributed to the control of parasitemia, cohorts of WT and PD-1KO mice were infected, then rested for 15 weeks after the clearance of the primary infection and then re-infected with *Pch*. On day -1 and every 2–3 days, mice were given either antibodies which block IFN-γ or control rIg. All WT mice (n = 7) developed parasitemia and blocking of IFN-γ had no significant effect on the course of the infection (Fig. 5b). In contrast, only 1/6 PD-1KO mice treated with rIg showed low level parasitemia around day 6–7 (Fig. 5c), while 3/6 PD-1KO mice depleted of IFN-γ following re-infection had detectable parasitemia levels as high as WT mice. Taken together these data show for the first time that PD-1 impedes protection by long-lived CD8^+^ T cell responses, and IFN-γ contributes to protection in some mice. Most significantly, while antibodies and CD4^+^ T cells can contribute to overall control of parasitemia during re-infection, CD8^+^ T cells are essential for control at sub-patent levels.

## Discussion

Even the best malaria vaccine candidate RTS/s, which will soon be licensed, has an efficacy of only 16.8% over a 4-year period^3^, highlighting our incomplete understanding of what it takes to mediate long term immunity against malaria. In a previous study, we showed a PD-1-mediated loss of numbers and functional capacity of parasite-specific CD8^+^ T cells during the acute phase of *Pch* malaria, which exacerbated acute infections and caused chronic disease^13^. We thus investigated if this loss of immunity during acute malaria could impact on long-term immunity by comparing responses to re-infection in WT and PD-1KO mice; 15weeks after the original infection had cleared. This study found that the bulk of parasitemia was controlled in both groups on re-infection. However, we also found that parasite-specific CD8^+^ T cells contributed to the control of parasitemia on re-infection in PD-1KO but not in WT mice, even when parasite-specific antibodies and CD4^+^ T cells were present in both groups. Thus, WT mice have losses in the functional capacity of CD8^+^ T cells during the acute phase of malaria^13^ which then prevents complete protection against new infections.

We undertook these studies as PD-1 has been implicated in the loss of immunity against primary malarial infections^11^,^12^,^13^,^14^,^15^,^22^, but the effects on memory responses were unknown. We used PD-1KO mice and a model of chronic malaria (*Pc*) which have recrudescent infections for 2.5 to 3 months after primary infection^10^. We had previously found no difference in parasite-specific antibody titers between WT and PD-1KO mice at the end of acute malaria (5 weeks) although PD-1KO mice developed sterile immunity while WT mice showed delayed clearance of parasitemia^13^. However, after 15 weeks, WT mice had higher parasite-specific antibody titers prior to challenge most likely due to recrudescent infections re-activating MBC responses^10^. Most importantly, we did find that while CD4^+^ T cell responses were relatively well maintained as previously reported^19^, PD-1 mediates a loss of numbers and functional capacity of CD8^+^ T cells during acute malaria which impacts on protection against re-infection.

The significance of CD8^+^ T cells in long term protection was highlighted by depletion studies. For this, we infected WT and PD-1KO mice with *Pc*, rested the mice for 15 weeks, and depleted CD8^+^ and CD4^+^ T cells compared to control mice given rIg, before all mice were re-infected. There were no differences in parasitemia in WT mice, with or without CD8^+^ T cell depletion. In contrast, 14% of PD-1KO mice treated with rIg had detectable parasitemia on re-infection compared to 86% of PD-1KO mice depleted of CD8^+^ T cells. This indicated the contribution of CD8^+^ T cells to long term immunity in PD-1KO mice. However, depletion of CD4^+^ T cells in PD-1KO mice had no significant effect indicating limited contribution of these cells to long-term protection.

It is widely perceived that immunity to malaria is, to an extent, defective and that one component of this defective immune response is the inability to induce or maintain long-term memory responses. If true, this would explain problems with the development of an effective vaccine against malaria. In areas with stable transmission of *Pf* parasites, even partially-protective immunity to malaria is acquired only after years of exposure and several infections. It has thus been speculated that malaria parasites are directly able to undermine the establishment and maintenance of immunological memory. It has also been suggested that long-lived antibody responses are not prerequisite for protection, and that antibody longevity varies in an exposure- and age-dependent manner^23^. In relation to long-lived T cell responses, previous studies in viral models have shown that effector CD8^+^ T cells can differentiate into long-lived protective memory T cells capable of self-renewal and rapid recall responses^24^. We thus propose that loss of effector CD8^+^ T cells during acute malaria as we have previously established^13^, contributes to a loss of long-lived protective memory CD8^+^ T cells capable of rapid expansion in response to new infections as highlighted in Fig. 4a.

CD8^+^ T cells are not generally considered as a target for blood stage malaria as mature red cells do not express MHC class I molecules. However, a growing body of evidence by different groups indicate that CD8^+^ T cell do play a role in protection against blood stage malaria^25^,^26^,^27^,^28^,^29^. Both *Pf* and *Pv* can also infect erythroblasts^30^,^31^ and recent studies found CD8^+^ T cells induce the externalization of phosphatidylserine on the infected erythroblasts by direct contact, which enhances the engulfment of the infected erythroid cells by phagocytes^32^. Alternatively, the role of CD8^+^ T cells may be indirect, in that the IFN-γ produced by these cells is required to maintain protective immunity by CD4^+^ T cells^33^. In conclusion, we now show that CD8^+^ T cells have a role in clearance of blood stage parasites, following long-term re-infection, which requires consideration in the design of future malaria vaccines.

## Methods and Materials

### Mice

Specific pathogen-free C57BL/6 (WT) mice were obtained from the Animal Resources Centre (Perth, Australia). PD-1KO (*Pdcd1*^−/−^) mice on a C57BL/6 background were kindly provided by Dr. T. Honjo through the Riken BRC^34^. PD-1KO and WT mice were housed in the QIMR animal research facility, and all procedures were approved and monitored by the QIMR Animal Ethics Committee. Work was conducted under QIMR animal ethics approval number A0209–622 M in accordance with the “Australian code of practice for the care and use of animals for scientific purposes” (Australian National Health & Medical Research Council).

### Parasitic infection and monitoring

Cohorts of WT or PD-1KO mice were inoculated intravenously with 10^5^ parasitized red blood cells (pRBC) freshly obtained from C57BL/6 mice previously infected with *P. chabaudi chabaudi* AS clone (*Pch*) as previously described^13^. Mice were monitored daily for, anemia, and physical symptoms of disease, including posture (hunching), lack of activity and fur texture. Mice were euthanized if they showed signs of significant distress. For re-infections, mice were also given 10^5^ pRBC and monitored for at least 10 days or until PD-1KO mice cleared the infection. For studies of sterile immunity, we transferred packed red cells from 200 μl blood taken from re-infected for PD-1KO mice into naïve C57Bl/6 mice.

### ELISA

Antibody titers were determined as previously described using ELISA plates coated with 5 or10 μg/ml *Pch* parasite antigen as determined for each batch. Curves of absorbance against serum dilution were plotted and the reciprocal dilution of the end titer determined based on the absorbance of serum from naive mice.

### ELISPOT assay for antibody-producing cells. 

Multiscreen-HA plates were coated with 5 or10 μg/ml *Pch* parasite antigen as determined for each batch. Isolated spleen or BM cells were directly tested for antibody secreting cells using a published method^35^,^36^.

### Flow cytometry on spleen cells

All antibodies were titrated for optimal staining prior to experimentation. The following antibodies were used for surface staining (obtained from Biolegend unless otherwise indicated): anti-CD3-PerCP-eFluor710 (17A2; eBioscience), anti-CD19-PE-Vio770 (6D5; Miltenyi Biotec), anti-CD4-Pacific Blue (GK1.5), anti-CD8α-VioGreen (53–6.7; Miltenyi Biotec), anti-CD8α-FITC (53–6.7), anti-PD-1-PE (RMP1-30), anti-Ki-67-FITC (SolA15; eBioscience) and anti-CD62L-biotin (MEL-14; BD Pharmingen). Viability was determined in some samples using Fixable Viability Dye eFluor780 (eBioscience). For identification of IFN-γ or IL-10 secreting cells by flowcytometry, the MACS Cytokine Secretion Assay (Miltenyi Biotec) was used. Streptavidin conjugated antibodies were detected using biotinylated PE or DyeLight 488 (Biolegend). The samples were analyzed on a BD FACSCanto or LSR Fortessa flow cytometer (BD biosciences, San Jose, CA, USA) (by gating on viable cells and using FCS express software version 3.3 (DeNovo Software, Los Angeles, CA, USA). Approximately between 10^4^ and 2 × 10^5^ cells were analyzed from each sample.

### *In vivo* depletion of CD4^+^ and CD8^+^ T cells

For the depletion of CD4^+^ or CD8^+^ T cells, intra-peritoneal injections of anti-CD4 antibody (500 μg; GK-1.5; Bio X cell), anti-CD8β antibody (500 μg; 53.5.8; Bio X cell) or control rat IgG (500 μg; Bio X cell) were administered on day −1 and then every 3–4 days for 14 to 18 days. Mice were infected on day 0. Notably, CD8^+^ T cells express CD8α and β chains while CD8^+^ DCs express two CD8α chains. As such, depletion of CD8β^+^ cells only depletes CD8^+^ T cells.

### Tetramer labeling

Parasite-specific F4 epitopes (H-2 K^b^ restricted) previously described for *P. berghei* ANKA^37^ and *P. chabaudi*^13^, were used as previously described^13^.

### ELISPOT assay to measure peptide-specific IFN-γ production

CD8^+^ T cells were isolated from *P. chabaudi*-infected mice, and dendritic cells were isolated from naive mice (for use as antigen-presenting cells), using a Dynabeads Untouched Mouse T cells or DC kit (Life Technologies) and then immuno-magnetically isolated using anti-mouse CD4, CD8 or CD11c Micro Beads (Miltenyi Biotec).

PVDF micro plates (Millipore, Bedford, MA, USA) were coated according to the manufacturer’s directions using the Mouse IFN-γ ELISPOT Ready-SET-Go kit (eBioscience). Approximately 2 × 10^5^ T cells, from individual mice, were co-cultured with 2 × 10^4^ DC, in 8–12 wells per per sample in these plates with 20 μg/ml peptide F4 or no peptide as previously described^38^. After 36 hours, the plates were processed according to the manufacturer’s instructions to quantify parasite-specific IFN-γ secretion.

### Statistics

The error bars for parasitemia are shown as the mean ± standard deviation. Assays with replicate wells from individual mice (biological replicates) are shown as Standard error of the mean (SEM). *P* values were determined using the non-parametric Mann-Whitney *U* test on pooled data from replicate experiments, analyzed using GraphPad Prism 5 (GraphPad Software).

## Additional Information

**How to cite this article**: Horne-Debets, J. M. *et al*. Mice lacking Programmed cell death-1 show a role for CD8^+^ T cells in long-term immunity against blood-stage malaria. *Sci. Rep.* **6**, 26210; doi: 10.1038/srep26210 (2016).
